# Supplementary material for: Breaking Barriers: Candidalysin Disrupts Epithelial Integrity and Induces Inflammation in a Gut-on-Chip Model
Source: Toxins (Basel). 2025 Feb 14;17(2):89. doi: 10.3390/toxins17020089 (PMC11861147; doi:10.3390/toxins17020089)
Supplement: Supplementary file 1 [file toxins-17-00089-s001.zip › toxins-3462118-supplementary.pdf]

# Supplementary Materials

Table 1. Table of reagents

| **Reagent** | **Supplier** | **Reference number** | **Note** |
|---|---|---|---|
| Candidalysin | Protein Research Ltd. (Fareham, UK) | N/A (custom peptide synthesis) | Sequence: SIIGIIMGILGNIPQVIQIIMSIVKAFKGNK<br>MW: 3310.11 Da (3.31 kDa) |
| Fluorescein sodium salt | Sigma-Aldrich, (St. Louis, MO, USA) | F6377-100G | Stock: 1mg/ml in water<br>Working: 10 µg/ml |
| TRITC-Dextran 4.4 kDa | Sigma-Aldrich, (St. Louis, MO, USA) | T1037 | Stock: 25mg/mL in water<br>Working: 500 µg/ml |
| ActinGreen™ 488 ReadyProbes™ Reagent | Sigma-Aldrich, (St. Louis, MO, USA) | R37110 | 2 drops/ml |
| NucBlue™ Live ReadyProbes™ Reagent | Sigma-Aldrich, (St. Louis, MO, USA) | R37605 | 2 drops/ml |
| DRAQ7™ Dye | Biostatus (Shepshed, UK) | DR71000 | Working: 3 µM |
| Triton 100X | Sigma-Aldrich, (St. Louis, MO, USA) | T8787 | |

<table>
<tr><td>Formaldehyde</td><td>Sigma-Aldrich, (St. Louis, MO, USA)</td><td>252549</td><td></td></tr>
<tr><td>HBSS</td><td>ThermoFisher (Waltham, MA, USA)</td><td>14025-092</td><td></td></tr>
<tr><td>PBS</td><td>ThermoFisher (Waltham, MA, USA)</td><td>70013-016</td><td>Diluted 10X in milliQ</td></tr>
<tr><td>EMEM</td><td>ATCC (Manassas, VA, USA)</td><td>30-2003</td><td rowspan="4"><u>Caco-2 medium:</u><br>440mL EMEM<br>50mL FBS<br>5mL P/S<br>5mL NEAA</td></tr>
<tr><td>FBS</td><td>ThermoFisher (Waltham, MA, USA)</td><td>16140-071</td></tr>
<tr><td>P/S</td><td>ThermoFisher (Waltham, MA, USA)</td><td>15140-122</td></tr>
<tr><td>MEN NEAA</td><td>ThermoFisher (Waltham, MA, USA)</td><td>11140-050</td></tr>
<tr><td>PROCARTAPLEX 10 PLEX</td><td>ThermoFisher (Waltham, MA, USA)</td><td>PPX-10</td><td>Analytes:<br>• G-CSF (LOQ: 16.46 pg/mL)<br>• GM-CSF (LOQ: 1.79 pg/mL)<br>• IL-1 alpha (LOQ : 0.61 pg/mL)<br>• IL-1 beta (LOQ : 0.32 pg/mL)<br>• IL-6 (LOQ : 9.77 pg/mL)<br>• IL-8 (LOQ : 2.44 pg/mL)<br>• IP-10 (LOQ : 1.95 pg/mL)<br>• MCP-1 (LOQ : 1.22 pg/mL)<br>• MIP-3 alpha (LOQ: 1.95 pg/mL)<br>• S100A8/A9 (LOQ: 60.84 pg/mL)</td></tr>
</table>

| | | | |
|---|---|---|---|
| LDH-Glo™ Cytotoxicity Assay | Promega (Madison, WI, USA), | J2380 | LOQ: 0.5 mU/mL |
| Human LL-37(Antibacterial Protein LL-37) ELISA Kit | Elabscience (Houston, TX, USA) | E-EL-H2438 | LOQ: 1,56 ng/mL |
| OrganoReady Colon Caco-2 3-lane 40 | MIMETAS (Oegstgeest, Netherlands) | MI-OR-CC-01 | Use caco-2 medium |
| OrganoReady Colon Organoid 3-lane 64 | MIMETAS (Oegstgeest, Netherlands) | MI-OR-CORG-02 | Includes medium:<br>• OrganoMedium Colon Organoid-ARM Apical Recovery Medium<br>• OrganoMedium Colon Organoid-BRM Basolateral Recovery Medium<br>• OrganoMedium Colon Organoid-ACM Apical Culture Medium<br>• OrganoMedium Colon Organoid-BCM Basolateral Culture Medium |
